# Could CCI or FBCI Fully Eliminate the Impact of Curve Flexibility When Evaluating the Surgery Outcome for Thoracic Curve Idiopathic Scoliosis Patient? A Retrospective Study

**DOI:** 10.1371/journal.pone.0126380

**Published:** 2015-05-18

**Authors:** Changwei Yang, Xiaofei Sun, Chao Li, Haijian Ni, Xiaodong Zhu, Shichang Yang, Ming Li

**Affiliations:** 1 Department of Spine Surgery, Changhai Hospital, the Second Military Medical University, Shanghai, 200433, China; 2 Department of Orthopedics, 304 Hospital of the People’s Liberation Army, Beijing, 100048, China; 3 Department of Nerve Internal Medicine, The General Hospital of the People's Liberation Army, Beijing, 100853, China; 4 Department of Nerve Internal Medicine, Xijing Hospital, the Fourth Military Medical University, Xi’an, 710032, China; Van Andel Institute, UNITED STATES

## Abstract

**Purpose:**

To clarify if CCI or FBCI could fully eliminate the influence of curve flexibility on the coronal correction rate.

**Methods:**

We reviewed medical record of all thoracic curve AIS cases undergoing posterior spinal fusion with all pedicle screw systems from June 2011 to July 2013. Radiographical data was collected and calculated. Student t test, Pearson correlation analysis and linear regression analysis were used to analyze the data.

**Results:**

60 were included in this study. The mean age was 14.7y (10-18y) with 10 males (17%) and 50 females (83%). The average Risser sign was 2.7. The mean thoracic Cobb angle before operation was 51.9°. The mean bending Cobb angle was 27.6° and the mean fulcrum bending Cobb angle was 17.4°. The mean Cobb angle at 2 week after surgery was 16.3°. The Pearson correlation coefficient r between CCI and BFR was -0.856(P<0.001), and between FBCI and FFR was -0.728 (P<0.001). A modified FBCI (M-FBCI) = (CR-0.513)/BFR or a modified CCI (M-CCI) = (CR-0.279)/FFR was generated by curve estimation has no significant correlation with FFR (r=-0.08, p=0.950) or with BFR (r=0.123, p=0.349).

**Conclusions:**

Fulcrum-bending radiographs may better predict the outcome of AIS coronal correction than bending radiographs in thoracic curveAIS patients. Neither CCI nor FBCI can fully eliminate the impact of curve flexibility on the outcome of correction. A modified CCI or FBCI can better evaluating the corrective effects of different surgical techniques or instruments.

## Background

Adolescent idiopathic scoliosis(AIS) is a complex three-dimensional deformation of the spine. Surgical treatment has been demonstrated to be an effective way of controlling curve progression. Nowadays, the transpedicular screw system is currently widely used for surgical correction of AIS. It is reported in the literature that posterior correction and fusion with pedicular screws in the treatment of AIS can enhance the correction rate, though results vary between different studies [1–5]. However, most of these series case reports did not take preoperative flexibility into account. As is widely known, regardless of the instrument system used, patients with flexible curves have better correction rates than those with rigid curves. As a result, The corrective ability of different instruments used between groups are often difficult to compare [6].

Several researchers have attempted multiple approaches to eliminate the effect of flexibility when trying to evaluate surgical outcome. Vora et al [7] use CCI(Clinical Correction Index)—the ratio of the correction rate and preoperative flexibility—to eliminate the influence of preoperative flexibility when evaluating the correction ability of different instruments. Cheung et al [6,8] use FBCI(fulcrum bending correction index), which is calculated by dividing the correction rate by the fulcrum flexibility, to eliminate the difference in flexibility between groups for the purpose of comparing influences of other factors, such as the surgeon and instrumentation, on the surgical correction rate. Since then, many other groups [9,10] have used CCI or FBCI to evaluate the correction capacity of instrumentation in their reports.

However, to our knowledge, there are currently no studies to clarify if the CCI or FBCI could truly eliminate the influence of curve flexibility when evaluating the correct ability between different internal fixation apparatus, thus it remains unclear if these indexes can in fact eliminate the effect of preoperative flexibility when evaluating the outcome of curve correction. So in this study, we explore the correlation between curve flexibility and CCI or FBCI to find if there is an correlation between CCI or FBCI with curve flexibility, in other words to find whether CCI or FBCI can effectively eliminate the impact of curve flexibility when evaluating the corrective force during surgery.

## Materials and Methods

### Ethics

The protocol for this study was approved by the human ethic committee of Changhai Hospital, Second Military Medical University, Shanghai, China. Written informed consent was given by the participants included in this study. All patient data was anonymized and de-identified in a confidential manner. The data was exclusively used for this study and was not shared with other individuals.

### Patient population

A retrospective cohort study of the radiographs and medical records of 98 patients with thoracic idiopathic scoliosis who received surgical treatment in Changhai hospital betweenJune 2011 and July 2013 was performed. Patients received spinal release or had previous history of surgery and manipulations that could affect spinal flexibility were excluded from the study. A total of 60 patients who met the follow-up criteria were included in this study. The inclusion criteria for patients were diagnosis with thoracic curve AIS (classified as Lenke type 1–4). All patients underwent posterior correction and fusion surgery with all pedicle screws system under general anesthesia and controlled hypotension. The neutral vertebra was usually selected as the proximal instrumented vertebra, and the vertebra one above stable vertebra was usually selected as the lower instrumented vertebra. Coronal curve correction was performed using the combination of translation, rod rotation techniques and compression/traction maneuvers. Titanium round rods were used in in these cases. The average screw density{number of implants/(fusion segments×2)} was 0.7(ranging from 0.6–1.0). For patients with a BFI<30%, Ponte Osteotomy was performed around the apex area of the curve. Decortication of the posterior elements was performed after the correction, and a freeze-dried bone allograft was used for the posterolateral fusion.

### Data measurement and calculation

Preoperative standing posteroanterior, lateral, and supine lateral bending radiographies and fulcrum bending radiographies were performed routinely before surgery on all patients who underwent surgery. Standing posteroanterior and lateral photographs were evaluated at 2 weeks after surgery. All the data was collected by attending surgeons that have at least 3 year work experience on spine deformity department separately. For data show a 3% variance, a third senior surgeon was involved in decision. The average of the data collected were used. Radiographical analysis included coronal Cobb angle on standing posteroanterior and bending radiographs. BFR (bending flexibility rate), FFR (fulcrum flexibility rate), CR (correction rate), CCI and FBCI were calculated as follows:

BFR (%) = (Major curve Cobb angle—bending Cobb angle)/major curve Cobb angle×100%.

FFR (%) = (Major curve Cobb angle-furculum bending Cobb angle) /major curve Cobb angle×100%.

CR (%) = (Major curve Cobb angle—postoperative Cobb angle)/major curve Cobb angle×100%.

CCI = CR/ BFR

FBCI = CR/ FFR

### Statistical analysis

Clinical and demographic characteristics between groups were analyzed by student t test. The relationship between BFR, FFR and CR, CCI, FBCI was analyzed by Pearson correlation and linear regression analysis. P<0.05 was considered statistically significant. Curve estimation was used to generate the equation of M-CCI and M-FBCI.. Statistical Package for Social Science software 16.0 (SPSS Inc., Chicago, IL, USA) was used to perform the statistical analysis.

## Results

### Clinical and radiological results

Of the 98 patients reviewed, 60 met the inclusion criteria(Lenke 1:40, Lenke 2:8, Lenke 3:11, Lenke 4:1). The mean age was 14.7y (10-18y) with 10 males (17%) and 50 females (83%) in the cohort. The average patient Risser sign grade was 2.7. The mean thoracic Cobb angle before surgery was 51.9° (40°–100°, SD: 13.1°). The mean lateral bending Cobb angle was 27.6° (7–87°, SD: 15.3°) and the mean furculum bending Cobb angle was 17.4° (3°–67°, SD: 13.8°). The mean Cobb angle at 2 week after surgery was 16.3° (4°–60°,SD: 10.7°). There was no significant difference between the fulcrum bending Cobb angle and the postoperative Cobb angle, but the difference between the bending Cobb angle and the postoperative Cobb angle was significant. The mean BFR was 49.4% (13%–83%, SD: 16.2%), and the mean FFR was 69.3% (27%–93%, SD: 69.3%). The mean CR of the main thoracic curve was 70.2% (40%–91%, SD: 11.8%), and the mean CCI and FBCI were 1.59 and 1.04, respectively. The results are shown in Table 1.

**Table 1:** Demographic Characteristics and Clinical Features of the Subjects.

Variables	Mean	Range	SD	P
Age at surgery (yr)	14.7	10–18	1.9	
Risser sign	2.7	0–5	1.6	
Preoperative thoracic Cobb	51.9°	40°–100°	13.1°	
Preoperative lateral bending Cobb	27.6°	7°–87°	15.3°	P<0.01*
Preoperative fulcrum bending Cobb	17.4°	3°–67°	13.8°	P = 0.106**
Postoperative thoracic Cobb (1 week)	16.3°	4°–60°	10.7°	
BFR (%)	49.4	13–83	16.2	
FFR (%)	69.3	27–93	69.3	
CR (%)	70.2	40–91	11.8	
CCI	1.59	0.68–3.42	0.62	
FBCI	1.04	0.63–1.87	0.19	

* Paired-samples T test between preoperative lateral bending Cobb and postoperative thoracic Cobb (1 week)

**Paired-samples T test between preoperative fulcrum bending Cobb and postoperative thoracic Cobb (1 week)

### Regression analysis

**Fig 1:**
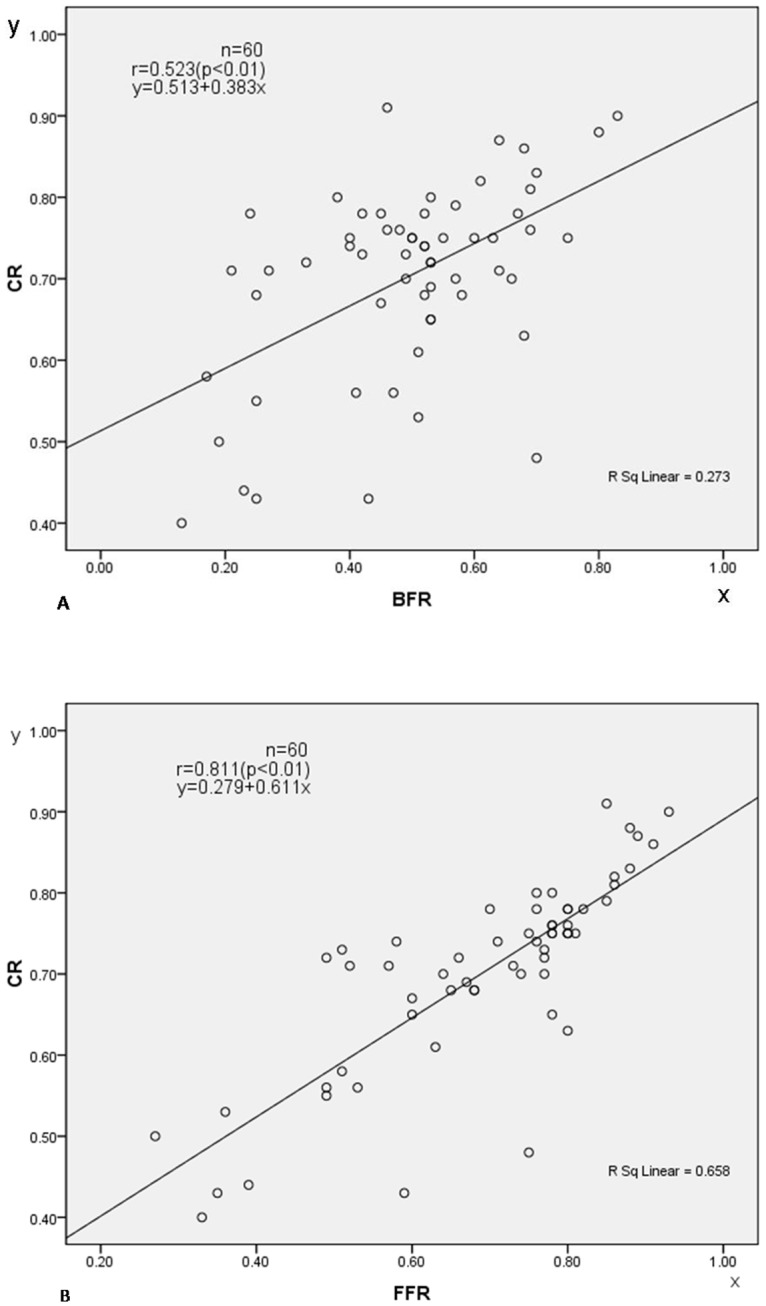
Show that FFR(r = 0.811) has a higher predict value than BFR(r = 0.523) in predicting CR. (A) scatter plot with correction rate (CR) against bending flexibility rate(BFR) and the linear regression equation of estimating CR by BFR. (B) scatter plot with correction rate (CR) against fulcrum flexibility rate (FFR) and the linear regression equation of estimating CR by FFR.

The Pearson correlation coefficient r between CR and BFR was 0.523(P<0.01) and between BFR and CCI was -0.856(P<0.01). The Pearson correlation coefficient r between CR and FFR was 0.811(P<0.01) while the r correlation coefficient between FFR and FBCI was -0.728(P<0.01). The linearregressionequation of estimating CR by BFR or FFR is CR = 0.383xBFR +0.513 or CR = 0.611xFFR +0.279 (Fig 1).

### Curve estimation

**Fig 2:**
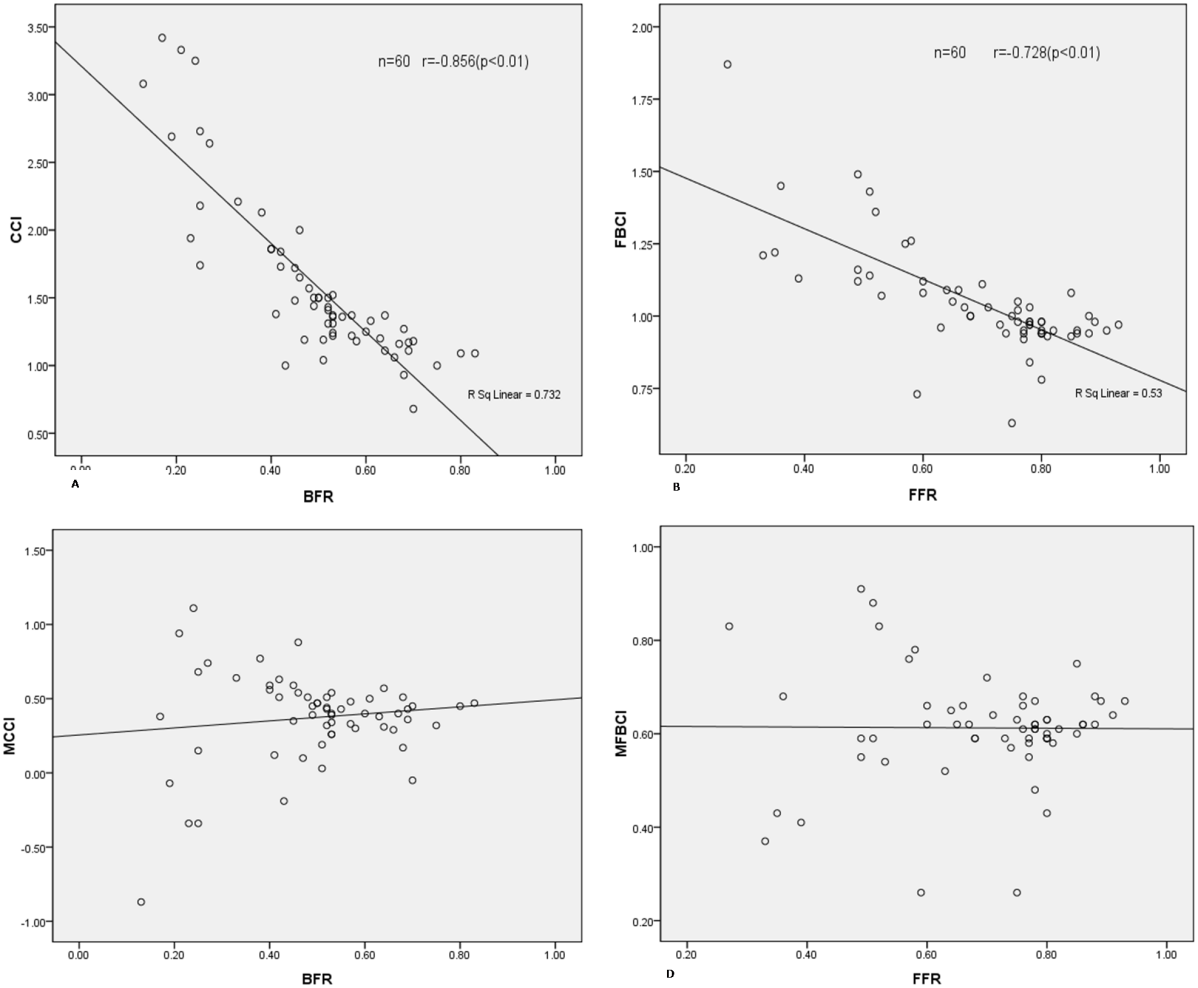
Correlation analysis between BFR (bending flexibility rate), FFR (fulcrum flexibility rate), CCI(correction index), fulcrum bending correction index (FBCI) modified FBCI (M-FBCI), modified CCI (M-CCI). A: CCI showed significant negative correlation with BFR(r = -0.856, P<0.01). B: FBCI showed significant negative correlation with FFR(r = -0.728, P<0.01). Figs 2A,2B show that both CCI and FBCI could not fully eliminate the influence of BFR and FFR. Means that the preoperative curve flexibility will still affect when using CCI or FBCI to compare the curve corrective ability of different apparatus. C: M- CCI showed no significant correlation with BFR(r = 0.123, P = 0.349). D: M-FBCI showed no significant correlation with FFR(r = -0.008, P = 0.950). Figs 2C,2D show that M-CCI and M-FBCI do not have significant correlation with preoperative curve flexibility. Means that the influence of curve flexibility could be eliminated when use these to compare the curve corrective ability of different apparatus.

The curve estimation show a modified FBCI (M-FBCI) = (CR-0.513)/BFR or a modified CCI (M-CCI) = (CR-0.279)/FFR can eliminate the influence of curve flexibility. Further linear regression shown there were no significant correlation between FFR and M-FBCI(r = -0.08, p = 0.950) or between BFR and M-CCI(r = 0.123, p = 0.349). The results are demonstrated in Table 2 and Fig 2.

**Table 2:** Results of Pearson Correlation Analysis.

Variables	BFR	FFR
CR	r = 0.523(P<0.01)	r = 0.811(P<0.01)
CCI	r = -0.856(P<0.01)	
FBCI		r = -0.728(P<0.01)
M- CCI	r = 0.123(P = 0.349)	
M-FBCI		r = -0.008(P = 0.950)

## Discussion

Preoperative assessment of curve flexibility is critical for selecting fusion segments and for predicting the degree of correction. Indeed, flexibility is an important parameter when estimating surgical correction. The flexibility of scoliosis can be assessed by various radiographs including lateral bending, fulcrum bending, traction, and push-prone films. When the King’s classification and single-plane correction instruments were used, bending films were generally accepted as the “golden standard” for predicting the flexibility of scoliosis [11]. However, with the development of third-generation instrumentation, curve correction has improved, and bending films no longer accurately reflect the outcome of surgical correction. Cheung and Luk [12] developed fulcrum-bending radiographs, during which the patient is in the lateral decubitus position and bent over a radiolucent padded cylinder, which acts as a fulcrum, so that the spine is passively hinged open. They found the fulcrum bending radiograph to be more predictive of flexibility and corrective ability than the lateral-bending radiograph in patients who had segmental spinal instrumentation for correction of idiopathic scoliosis. Since then, fulcrum radiographs have been used by many centers for preoperative assessment of curve flexibility. The result of our study also showed that there was no significant difference between the mean angles measured on the fulcrum bending radiograph (17°) and the postoperative standing radiograph of (16°, P = 0.106). However, there was a significant difference between the mean angle measured on the supine lateral bending radiograph (28°) and the postoperative standing radiograph of (16°, P<0.01), the linear regression of CR = 0.383xBFR +0.513; CR = 0.611xFFR +0.279 also suggesting that fulcrum radiographs can better predict the outcome of surgical correction than bending radiographs.

The term “correction rate” is used in describing the surgical effect of coronal correction of scoliosis [13–15]. The outcome of curve correction can be determined by multiple factors, including surgical technique, instruments, and curve flexibility. We excluded Lenke type C curves in this study because many surgeons deliberately undercorrect the curve in order to achieve good trunk balance after surgery. Although the correction rate can be affected by the curve flexibility, many studies does not take preoperative flexibility into account when comparing the corrective effects of surgery using different techniques or instruments [16,17]. The different correction rates reported in studies using different instruments may be due to differences in patient curve flexibility rather than the effects of the instruments themselves. In our previous study, we matched curve flexibility to eliminate its influence on the final correction rate; the results of this study suggested that the pedicle screw system had a better corrective effect when compared with the hybrid hook screw system [18]. Vora et al [7] defined the ratio between the scoliotic correction rate and preoperative flexibility as CCI, and used this index to eliminate the influence of curve flexibility when compare corrective ability of different instruments. FBCI is based on the same principle with the only difference being that FBCI uses fulcrum bending flexibility instead of bending flexibility. As CCI or FBCI take curve flexibility into account, they are more useful in describing curve correction outcomes—especially when comparing different case series. In a study using fulcrum-bending radiographs to compare thoracic curve correction using four different instruments, Luk et al [6] found that there were differences in the correction rate between these groups. While, when FBCI was used to analyze the result, all the instruments showed the same efficacy in AIS coronal curve correction.

Our current study suggests that CCI or FBCI could not fully eliminate the influence of preoperative flexibility when evaluating the correction capability of different instruments. In particular, our data showed that both CCI (r = -0.856) and FBCI (r = -0.728) have a strong negative relationship with preoperative flexibility. These results suggest that CCI and FBCI do not fully eliminate the impact of curve flexibility and that it is not that accurate to use these indexes to evaluate the correction efficiency of instruments—especially in patient groups with variability in curve flexibility. Cheung et al [19] also found that the FBCI decreased as the FFR increased and they postulated that this was because the pedicle screw system has a great correction efficiency when facing large, rigid curves. Luk et al [6] attributed this relationship to the surgeon—suggesting that it was because the correction force used during operation was greater than the corrective force from the body weight in the fulcrum-bending position in cases of rigid scoliosis. Our data shows that this negative relationship exists in both bending radiographs and fulcrum-bending radiographs and cannot be explained by surgical factors alone. Generally speaking, there is a negative relationship between CCI or FBCI and the preoperative flexibility index regardlessof whether the curve is flexible or rigid.

An innovator of our study was the modified CCI or FBCI. Equations of M-CCI = (CR-0.513)/BFR and M-FBCI = (CR-0.279)/FFR were given by curve estimation to generate a best curve in eliminating the influence of flexibility on correction rate. In another study by Luk et al [20], they defined the difference between the preoperative fulcrum bending angle and postoperative PA angle as AngleXF. Proposed the notion of “XFI”, calculated by divide the AngleXF to the FFR, to measure the curve corrective ability contributed by different factors such as instrument, surgeon and so on. However, XFI has a significant correlation to curve flexibility too, means it was not proper to use it to evaluate the corrective ability of different factors. In our study, the M-FBCI (modified FBCI) has no significant correlation(r = -0.08, p = 0.950) with FFR and M-CCI (modified CCI) has no significant correlation with BFR (r = 0.123, p = 0.349), this suggests that the M-FBCI or M-CCI was not affected by preoperative curve flexibility and was better in evaluating the corrective capability of the different surgical techniques or instruments than the standard FBCI and CCI.

One potential concerns of our study might be the sample size. Though not very large, a sample size of 60 consecutive thoracic AIS patients is enough to build a liner regression [21,22]. Another concern of our study might be the variation between different centers. Actually, the constant (in our study, 0.513 or 0.279) may vary slightly among different spine centers due to the differences in surgical techniques, bending radiographies, or furculum bending radiographies. But these take together only has limited affection to the equation of M-FBCI or M-CCI, the overall trend will be unchanged. Taken together, M-FBCI and M-CCI give us a more precise way to evaluate the correction ability of different internal fixation systems in thoracic curve AIS patients. More studies need to be done in other Lenke type AIS patients.

## Conclusions

The data in this study showed that fulcrum bending radiographs can better predict the outcome of AIS coronal correction than bending radiographs in correction of thoracic curve in cases using transpedicular screw system. Both CCI and FBCI can not fully eliminate the impact of curve flexibility on the outcome of correction. A modified CCI or FBCI is needed when evaluating the corrective effects of the different surgical techniques or instruments.

## Supporting Information

S1 Data (XLSX)
